# Genetic scores based on risk-associated single nucleotide polymorphisms (SNPs) can reveal inherited risk of renal cell carcinoma

**DOI:** 10.18632/oncotarget.7623

**Published:** 2016-02-23

**Authors:** Yishuo Wu, Ning Zhang, Kaiwen Li, Haitao Chen, Xiaolin Lin, Yang Yu, Yuancheng Gou, Jiangang Hou, Deke Jiang, Rong Na, Xiang Wang, Qiang Ding, Jianfeng Xu

**Affiliations:** ^1^ Department of Urology, Huashan Hospital, Fudan University, Shanghai, PR China; ^2^ Fudan Institute of Urology, Huashan Hospital, Fudan University, Shanghai, PR China; ^3^ Department of Urology, Sun Yat-sen Memorial Hospital, Sun Yet-sen University, Guangdong, PR China; ^4^ State Key Laboratory of Genetic Engineering, School of Life Sciences, Fudan University, Shanghai, PR China; ^5^ Center for Genetic Epidemiology, School of Life Sciences, Fudan University, Shanghai, PR China; ^6^ Health Communication Institute, School of Public Health, Fudan University, Shanghai, PR China; ^7^ Program for Personalized Cancer Care, NorthShore University HealthSystem, Chicago, IL, USA

**Keywords:** renal cell carcinoma, SNPs, genetic score, Chinese

## Abstract

The objective of this study was to evaluate whether renal cell carcinoma (RCC) risk-associated single nucleotide polymorphisms (SNPs) could reflect the individual inherited risks of RCC. A total of 346 RCC patients and 1,130 controls were recruited in this case-control study. Genetic scores were calculated for each individual based on the odds ratios and frequencies of risk-associated SNPs. Four SNPs were significantly associated with RCC in Chinese population. Two genetic score models were established, genetic score 1 (rs10054504, rs7023329 and rs718314) and genetic score 2 (rs10054504, rs7023329 and rs1049380). For genetic score 1, the individual likelihood of RCC with low (<0.8), medium (0.8-1.2) and high (≥1.2) genetic score 1 was 15.61%, 22.25% and 33.92% respectively (P-trend=6.88×10^−7^). For genetic score 2, individual with low (<0.8), medium (0.8-1.2) and high (≥1.2) genetic score 2 would have likelihood of RCC as 14.39%, 24.54% and 36.48%, respectively (P-trend=1.27×10^−10^). The area under the receiver operating curve (AUC) of genetic score 1 was 0.626, and AUC of genetic score 2 was 0.658. We concluded that genetic score can reveal personal risk and inherited risk of RCC, especially when family history is not available.

## INTRODUCTION

Accounting for 2% of all malignancies, kidney cancer has become one of the most important health problems worldwide, with an estimated 270,000 new cases and 116,000 deaths every year. [1] Renal cell carcinoma (RCC) represents about 90% of all kidney cancer, and clear cell RCC (ccRCC) is the most common type in adults. Radical resection is considered as a curative treatment for early stage RCC. However, with the lack of diagnostic screening biomarkers, early diagnosis of RCC is challenging.

Besides the environmental risk factors (e.g., smoking, obesity, hypertension, etc.), inherited risk has been considered as one of the most important risk factors of RCC. Risks of sporadic RCC will increase more than two folds if individuals have a first-degree relative with RCC. [2, 3] Therefore, risk factors consultant (for both environmental and inherited) is important for evaluating individual risk of RCC. Family history can reflect inherited risk; however, the accuracy of family history can be affected by the family size, member age, etc. In addition, family history is uninformative in China because of the health policy in the past decades. To address this problem, genetic score is considered as one of the useful tools to measure inherited risks of diseases. [4]

In this study, we evaluated whether genetic scores based on RCC risk-associated single nucleotide polymorphisms (SNPs) could reflect the individual inherited risk of RCC in Chinese population.

## RESULTS

Baseline characteristics of the study population were summarized in Table 1. All candidate SNPs of control group passed the test of Hardy-Weinberg equilibrium and were polymorphic in Chinese population. Among the 10 SNPs, rs7023329 (odds ratio, OR=0.60, 95% confidence interval, 95%CI: 0.50-0.72, P=1.91×10^−8^) was significantly associated with ccRCC in Chinese population (Table 2). Combining with the results from previous study [5], 4 SNPs were significantly associated with RCC risk in Chinese population, and were used to establish the genetic risk models (Table 3). Two genetic risk models were finally established. Genetic score 1 was based on rs10054504, rs7023329 and rs718314, and we use rs1049380 to substitute for rs718314 in genetic score 2, because rs718314 and rs1049380 had relatively strong linkage disequilibrium (LD, R^2^=0.58) which might bring duplication efforts to the model if we would include both of them.

**Table 1:** Characteristics of study subjects

Variables	Case	Control	P-value	P-value^c^
N of cases	346	1130	-	-
Age (mean±SD)^a^	55.9±12.5	51.2±9.7	0.0001	-
Gender, # (%)			0.52	-
Male	230 (66.5)	732 (64.8)	-	-
Female	114 (32.9)	398 (35.2)	-	-
Location of Tumor, # (%)				
Left Kidney	155(46.5)	-	-	-
Right Kidney	158 (47.4)	-	-	-
Missing	20 (6.0)	-	-	-
Tumor Size (mean±SD)	3.63±2.42	-	-	-
Genetic Score				
Genetic score 1 (median, quartiles) ^b^	1.13 (0.80-1.36)	0.80 (0.57-1.26)	9.09×10^−13^	7.34×10^−11^
Genetic score 2 (median, quartiles) ^b^	0.97 (0.71-1.61)	0.76 (0.54-1.27)	3.66×10^−19^	1.49×10^−18^

a Age at diagnosis for cases or at recruitment for controls.

b Genetic score 1 is established based on rs10054504, rs7023329 and rs718314; genetic score 2 is established based on rs10054504, rs7023329 and rs1049380.

**Table 2:** Results of association test in Chinese population for reported RCC risk-associated SNPs from studies other than GWAS

Origin Popluation	Chr	SNP	Gene	Position	Minor/Major Alleles	ccRCC vs. Controls					
						F_A	F_U	OR	Lower 95%CI	Upper 95%CI	P-value
Caucasian	1	rs1326889	*AGT*	230862998	C/T	0.101	0.099	1.02	0.76	1.36	0.91
Caucasian	2	rs9679290	*EPAS1*	46557644	G/C	0.353	0.315	1.18	0.98	1.41	0.08
Caucasian	2	rs4953346	*EPAS1*	46558208	T/G	0.332	0.312	1.09	0.91	1.31	0.35
Caucasian	2	rs12617313	*EPAS1*	46559776	A/T	0.029	0.016	1.87	1.06	3.28	0.04^a^
Caucasian	3	rs1492078	*AGTR1*	148415177	T/C	0.186	0.183	1.02	0.82	1.28	0.85
Chinese	6	rs2010963	*VEGFA*	43738350	C/G	0.375	0.409	0.87	0.73	1.04	0.12
Chinese	9	rs2279776	*PTPRD*	8389364	C/G	0.331	0.328	1.02	0.84	1.22	0.87
Caucasian	9	rs7023329	*MTAP*	21816528	G/A	0.36	0.490	0.60	0.50	0.72	**1.91×10^−8^**
Chinese	12	rs17512051	*RKIP*	118573667	A/T	0.094	0.087	1.09	0.81	1.46	0.59
Chinese	19	rs895819	*pre-miR-27a*	13947292	C/T	0.271	0.280	0.96	0.79	1.16	0.68

Abbreviations: F_A: Frequency of affected (Case); F_U: Frequency of unaffected (Control); OR: Odds ratio; CI: Confidence interval

a The P value was calculated by Fisher exact test.

**Table 3:** Summarization of significant risk SNPs for RCC in the study population which will be used for genetic scores

SNPs	Risk allele	OR	P-value
rs10054504^5^	T	1.41	3.82×10^−61^
rs7023329	A	1.67	1.91×10^−8^
rs718314^5^	G	1.79	5.26×10^−8^
rs1049380^5^	A	1.58	0.0025

Abbreviations: OR: Odds ratio; RCC: Renal cell carcinoma; SNP: Single nucleotide polymorphism

**Figure 1:**
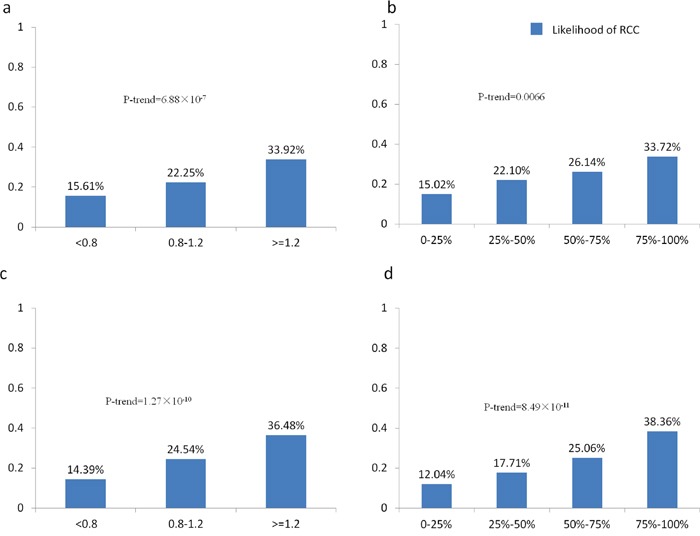
Likelihood of RCC with different genetic scores ranges in the study population **a.** likelihood of RCC among individuals with low (<0.8), medium (0.8-1.2) and high (≥1.2) genetic score (genetic score 1); **b.** likelihood of RCC among individuals with low, medium-low, medium-high and high (categorized by quartiles of genetic score) genetic score (genetic score 1); **c.** likelihood of RCC among individuals with low (<0.8), medium (0.8-1.2) and high (≥1.2) genetic score (genetic score 2); **d.** likelihood of RCC among individuals with low, medium-low, medium-high and high (categorized by quartiles of genetic score) genetic score (genetic score 2).

**Figure 2:**
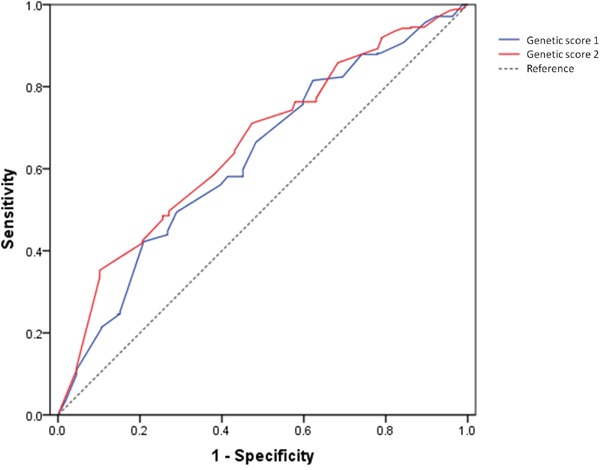
AUCs of genetic score 1 (blue, AUC=0.626, 95%CI: 0.593-0.660) and genetic risk 2 (red, AUC=0.658, 95%CI: 0.625-0.692) for predicting RCC risk

The median of genetic score 1 was significantly higher in RCC group than in control group (1.13 in RCC vs. 0.80 in control, P=9.09×10^−13^. Table 1). This remained significant when adjusting for age by logistic regression (P=7.34×10^−11^). The likelihood of RCC for individual who had low (<0.8), medium (0.8-1.2) and high (≥1.2) genetic score 1 was 15.61%, 22.25% and 33.92% respectively (P-trend=6.88×10^−7^, Figure 1a). The OR between high genetic score and low genetic score was 2.17. The study population was then stratified to four groups based on the quartiles of the genetic score. The likelihood of RCC for individual with low (0-25%th percentile), medium-low (25%th-50%th percentile), medium-high (50%th-75%th percentile) and high (75%th-100%th percentile) genetic score 1 was 15.02%, 22.10%, 26.14% and 33.72%, respectively (P-trend=0.0066, Figure 1b). Similarly, the median of genetic score 2 was significantly higher in RCC group than in control group (0.97 in RCC vs. 0.76, P=3.66×10^−19^). It remained significant when adjusting for age (P=1.49×10^−18^). Individual with low (<0.8), medium (0.8-1.2) and high (≥1.2) genetic score 2 would have likelihood of RCC as 14.39%, 24.54% and 36.48%, respectively (P-trend=1.27×10^−10^, Figure 1c). The OR between high genetic score and low genetic score was 2.54. Individual who had low (0-25%th percentile), medium-low (25%th-50%th percentile), medium-high (50%th-75%th percentile) and high (75%th-100%th percentile) genetic score 2 would have 12.04%, 17.71%, 25.06% and 38.36% likelihood of RCC (P-trend=8.49×10^−11^, Figure 1d). The area under the receiver operating curve (AUC of ROC) of genetic score 1 was 0.626 (95%CI: 0.593-0.660), and AUC of genetic score 2 was 0.658 (95%CI: 0.625-0.692) (Figure 2), indicating that both genetic score 1 and genetic score 2 could predict the RCC risk of individuals.

## DISCUSSION

To our knowledge, this is the first time we use risk-associated SNPs based genetic scores to evaluate personal risks for RCC in Chinese population. In the current study, our objective was to establish a tool to evaluate individual inherited risks of RCC supplemented to family history, just because of the uninformative family history in Chinese population due to the healthcare and birth control policy. We found that genetic scores based on risk SNPs could reveal the personal risk of RCC.

Studies suggested that family history was an important risk factor for RCC. [3, 6-8] An Icelandic research found that 60% of RCC patients had first or second degree family history. [6] Another comprehensive meta-analysis study suggested a more than 2 folds increase of risk for RCC if individual had first degree relatives having RCC, and the risk would increase by 4.3 folds if individual had any degree family history. [3] These indicated a strong inherited risk of RCC. In the current study, because of the uninformative family history in Chinese population (only 1 out of 346 patients in the present study had positive family history), we established genetic scores to reveal the inherited risks (the genetic scores of the only one case with positive family history was 2.11 for genetic score 1 and 1.61 for genetic score 2, indicating an increase of RCC risk than average level of population). We found that the risks of RCC for individuals having high genetic scores (≥1.2) would increase by ∼2.5 folds comparing to individuals with low genetic scores (<0.8). We thus indicated that genetic score was useful to evaluate inherited risk of developing sporadic RCC, and would have some potential clinical application. For example, individual with a high genetic score may add some imaging or ultrasound tests during annual health check in their 60s-70s; [9] for patients without family history, the genetic scores may provide inherited information; if a patient with a suspicious lesion in the kidney which cannot be diagnosed through the imaging features, genetic score may evaluate the probability of malignancy. However, further investigate is necessary to evaluate the application of genetic scores.

Genetic scores could provide more accurate assessment for inherited risks of cancers than family history. [4, 10, 11] Although family history do not require lab test, it will be influenced by member's age (disease not occurs), family size (negative family history due to lack of family member), recall biases (not able to be informed or remember the disease status of relatives), etc. In China, the situation goes to extremes. It is because of the poor healthcare policy (which limited the detection of diseases) in the past decades, the incomplete cancer registration in the past and the family planning policy (birth-control policy) limited the side of family (interfere the existence of affected siblings). Therefore, genetic scores may be more applicable and useful to evaluate personal inherited risk of RCC, especially in China.

Our study is not devoid of limitation: (1) the relatively small sample size of the study might challenge the power of our statistics even we have significant association results. Larger studies are needed to provide external validation and further evaluation between genetic scores and RCC. (2) The cutoff values used in this study were subjective by just using <0.8, 0.8-1.2, ≥1.2 or quartiles. However, we believed that they would be easier to understand. A genetic score of 0.8 means the risk for RCC of an individual is below average population risks, while a genetic score of 1.2 means the risk for RCC is higher than average. Efforts should be devoted to evaluate the clinical application of genetic scores.

In conclusion, Genetic score can reveal personal inherited risk of RCC, especially when family history is not available. Further investigation in large scale studies will be necessary to evaluate its potential application.

## MATERIALS AND METHODS

### Study population and study design

All patients with ccRCC (346 cases) were recruited from our institute (Huashan Hospital, Fudan University, Shanghai, China) from 2010 to 2014. A total of 1,130 health people from community populations in Shanghai were included as control group. The inclusion/exclusion criteria and the characteristics of the population from case and control groups were described reported in the previous studies. [5, 12] Briefly, in case group, patients with sporadic and surgical resectable kidney tumors, diagnosed as ccRCC by pathologists, were included. Written informed consent was obtained from each patient. The study was approved by institutional review board of Huashan Hospital, Fudan University, Shanghai, China.

In the previous study, we evaluated all established or potential RCC risk-associated SNPs from published Caucasian/African-American based genome-wide association study (GWAS) in Chinese population (the SNPs which were significantly associated with ccRCC in the previous study were summarized in Table 3). [5] In the current study, reported RCC risk-associated SNPs from studies other than GWAS were evaluated. Among 10 SNPs, 6 SNPs were from Caucasian based studies and 4 SNPs were from Chinese population based studies. (Table 4) [13-20].

**Table 4:** Reported RCC risk-associated SNPs from studies other than GWAS in different population

Chr	References	Origin population	Study Type	SNP	Gene	Position
1	Gabriella Andreotti, et al.^13^	Caucasian	Candidate SNPs	rs1326889	*AGT*	230862998
2	Summer S Han, et al.^14^	Caucasian	Fine mapping	rs9679290	*EPAS1*	46557644
2	Summer S Han, et al.^14^	Caucasian	Fine mapping	rs4953346	*EPAS1*	46558208
2	Summer S Han, et al.^14^	Caucasian	Fine mapping	rs12617313	*EPAS1*	46559776
3	Ivette A. Deckers, et al.^15^	Caucasian	Candidate SNPs	rs1492078	*AGTR1*	148415177
6	Chao Qin, et al.^16^	Chinese	Candidate SNPs	rs2010963	*VEGFA*	43738350
9	Yan Du, et al.^17^	Chinese	Candidate SNPs	rs2279776	*PTPRD*	8389364
9	F Gu, et al.^18^	Caucasian	Candidate SNPs	rs7023329	*MTAP*	21816528
12	Qiang Cao, et al.^19^	Chinese	Candidate SNPs	rs17512051	*RKIP*	118573667
19	Danni Shi, et al.^20^	Chinese	Candidate SNPs	rs895819	*pre-miR-27a*	13947292

Abbreviations: RCC: Renal cell carcinoma; GWAS: Genome-Wide Association Studies.

Blood Samples were collected from each study population. Whole-genome DNA was extracted using Puregene DNA Purification Kit (for case group) and Qiagen QIAamp DNA Blood Mini Kit (for control group). SNPs genotyping were performed using MassARRY iPLEX system (Sequenom Inc., San Diego, CA) at Fudan University, Shanghai, China. Four subjects and four negative controls (water samples) were duplicated in the 384-well panel for quality control. All the control samples were previously performed GWAS by using Illumina Human OmniExpress Bead Chips. SNPs were imputed by using IMPUTE 2.2.2 program based on haplotype data from the 1000 Genomes Project CHB+JPT population (Phase I integrated data version 3, released March 2012), because these SNPs were not included in the GWAS chip. All assays were performed in blinded fashion.

### Statistic analysis

All the SNPs were evaluated for Hardy-Weinberg equilibrium. The association between SNPs and ccRCC were analyzed by logistic regression, or Fisher's exact test if the allele frequency of SNP was lower than 5%. The analysis was performed by using PLINK 1.09 with 2-tailed P values of 0.005 (after Bonferroni correction P=0.05/10).

For each individual in the study, a genetic score was calculated based on personal genotype of the SNPs which are significantly associated with RCC and weighted by odd ratios (ORs): (1) the allelic OR for each SNP was obtained from our studies based on Chinese population; (2) the genotypic OR was calculated assuming a multiplicative model of allelic OR (for example, carrying two risk alleles=OR^2^, carrying one risk allele=OR, not carrying risk allele=1); (3) the relative risk to average risk level in Chinese population was calculated based on genotypic OR and genotype frequency (HapMap CHB population data); (3) the genetic score was calculated by multiplying relative risk of each SNPs based on different genotype of each individual (a sample is shown in Supplementary Material).

T-test (for normal distribution values) and Mann-Whitney U-test (for non-normal distribution values) were used to evaluate the differences of variables between groups. Chi-square trend tests and ROC analyses were used to analyze the prediction ability of genetic scores. These statistic analyses were performed by using SPSS 19.0. In this part, two-tailed P values of 0.05 were considered significant.

## SUPPLEMENTARY MATERIAL


