# Supplementary material for: Genetic scores based on risk-associated single nucleotide polymorphisms (SNPs) can reveal inherited risk of renal cell carcinoma
Source: Oncotarget. 2016 Feb 23;7(14):18631–7. doi: 10.18632/oncotarget.7623 (PMC4951315; doi:10.18632/oncotarget.7623)
Supplement: Supplementary file 1 [file oncotarget-07-18631-s001.pdf]

SUPPLEMENTARY MATERIAL

Method of calculating genetic score

Sample

1. Assuming a SNP1: C/T
   Allelic OR of T=1.37, Frequency of allele T=0.28
2. Genotype relative risk (RR)
   RR-TT=1.37^2=1.88; RR-CT=1.37, and RR-CC=1.0
3. Average population risk for the SNP1
   W=0.28^2×RR-TT+2×0.28×(1-0.28)×RR-CT+$(1\text{-}0.28)^2$×RR-CC=1.22
4. Risk relative to the general population (RRpop)
   RRpop-TT=1.88/1.22=1.54, RRpop-CT=1.37/1.22=1.12, RRpop-CC=1/1.22=0.82
5. Overall risk relative to the population for several SNPs
   Genetic score= RRpop1 × RRpop2 × RRpop3....
